# A Multi-Omics Approach Reveals Interleukin 1 Beta Priming as a Key Driver of Immunomodulatory and Regenerative Programs in Adipose-Derived Stem Cells for Osteoarthritis Therapy

**DOI:** 10.3390/cells15121056

**Published:** 2026-06-09

**Authors:** Vitale Miceli, Mattia Emanuela Ligotti, Vincenzo Raffo, Silvia Lopa, Viviana Ippolito, Alessia Gallo, Nicola Cuscino, Simone Dario Scilabra, Margot Lo Pinto, Simone Messina, Salvatore D’Arpa, Matteo Moretti, Laura de Girolamo, Matteo Bulati, Alessandra Colombini

**Affiliations:** 1 Department of Research, IRCCS ISMETT (Istituto Mediterraneo per i Trapianti e Terapie ad Alta Specializzazione), 90127 Palermo, Italy; vmiceli@ismett.edu (V.M.); agallo@ismett.edu (A.G.); smessina@ismett.edu (S.M.); mbulati@ismett.edu (M.B.); 2 Dipartimento di Medicina di Precisione e Rigenerativa e Area Jonica (Di.Me.Pre-J), Università Degli Studi di Bari, Valenzano, 70124 Bari, Italy; v.raffo@phd.uniba.it; 3 Cell and Tissue Engineering Laboratory, IRCCS Galeazzi-Sant’Ambrogio Hospital, Via Cristina Belgioioso 173, 20157 Milan, Italy; 4 Proteomics Group of Ri.MED Foundation, Research Department, IRCCS ISMETT (Istituto Mediterraneo per i Trapianti e Terapie ad Alta Specializzazione), 90127 Palermo, Italy; 5 Plastic and Reconstructive Surgery and Breast Unit, La Maddalena Cancer Center, 90146 Palermo, Italy; 6 Regenerative Medicine Division, Institute for Translational Research, Università della Svizzera Italiana (USI)–Ente Ospedaliero Cantonale (EOC), 6500 Bellinzona, Switzerland; 7 Service of Orthopaedics and Traumatology, Department of Surgery, Ente Ospedaliero Cantonale (EOC), 6900 Lugano, Switzerland; 8 Euler Institute, Faculty of Biomedical Sciences, Università della Svizzera Italiana (USI), 6900 Lugano, Switzerland; 9 Orthopaedic Biotechnology Laboratory, IRCCS Galeazzi-Sant’Ambrogio Hospital, 20157 Milan, Italyalessandra.colombini@grupposandonato.it (A.C.)

**Keywords:** adipose derived mesenchymal stem cells, interleukin 1 beta priming, multi-omics, immunomodulation, cartilage remodeling, osteoarthritis

## Abstract

**Highlights:**

**What are the main findings?**

- IL1β priming induces strong multi-omics reprogramming of adipose-derived mesenchymal stem cells compared to hypoxia and IFNγ.
- IL1β-primed ASCs display coordinated transcriptional and secretory signatures, including proteins and exosomal miRNAs, associated with immunomodulation and cartilage remodeling.

**What are the implications of the main findings?**

- IL1β priming may enhance the therapeutic potential of ASCs for osteoarthritis treatment.
- Multi-omics integration provides mechanistic insight and candidate targets for optimizing ASC-based regenerative strategies.

**Abstract:**

Osteoarthritis is a chronic degenerative joint disease characterized by inflammation and cartilage degradation, for which current treatments are mainly symptomatic and unable to halt disease progression. Adipose-derived mesenchymal stem cells (ASCs) represent a promising therapeutic option due to their regenerative and immunomodulatory properties, which may be further enhanced through specific priming strategies. In this study, primary human ASCs were exposed to interleukin-1 beta (IL1β), interferon-gamma (IFNγ), or hypoxic priming, and subsequently analyzed using a multi-omics approach integrating RNA sequencing, proteomics of secretome, and exosomal miRNA profiling. Differential gene expression, protein abundance, and miRNA signatures were assessed together with functional enrichment and network analyses. IL1β priming induced marked transcriptional reprogramming of ASCs, while hypoxia and IFNγ priming produced limited changes. IL1β also profoundly reshaped the ASC secretome and exosomal miRNA cargo, revealing coordinated regulation of pathways involved in immune modulation and cartilage remodeling. In contrast, the other priming conditions showed minimal and less integrated molecular effects. Overall, IL1β priming consistently generated a multi-layered molecular signature linking immunoregulatory and regenerative pathways. These findings suggest that IL1β priming enhances the functional properties of ASCs and provides mechanistic insight supporting their potential use in osteoarthritis therapy.

## 1. Introduction

Osteoarthritis (OA) is a chronic, disabling joint disease [1,2,3] caused by traumatic or age-related degenerative processes that trigger inflammatory and catabolic responses and lead to long-term joint destruction [4,5,6]. Traditional OA conservative management is based on symptomatic pharmacological treatments, which are ineffective in reversing or slowing disease progression and are characterized by side effects, especially when administered repeatedly over time [7,8]. Intra-articular injection of adipose-derived mesenchymal stem cells (ASCs) represents a promising and convenient approach for OA treatment given their regenerative and immunomodulatory properties [9,10,11]. ASCs are a population of mesenchymal stromal/stem cells (MSCs) derived from adipose tissue, sharing the defining biological features of MSCs while retaining tissue-specific properties [12,13,14]. MSCs are able to secrete growth factors, cytokines, chemokines, and extracellular vesicles (EVs), which modulate fibrosis, immune responses, angiogenesis, and the activation of tissue-resident stem cells, thereby orchestrating tissue regeneration and functional recovery [15,16,17]. Due to their features, ASC-based products are effective in improving patients’ conditions, and their benefits could be further enhanced by modulating their biological potential as needed. MSCs are highly responsive to their surrounding microenvironment, such as the osteoarthritic milieu, and adapt their behavior accordingly. For this reason, inflammatory priming represents an innovative strategy to obtain ASCs with increased potency. In fact, ASCs respond to priming with OA synovial fluid [18] or with interleukin1-β (IL1β) [19,20,21] in an adaptive way by tuning their immunomodulatory potential. Treatment with IFN-γ promotes the acquisition of an immunomodulatory phenotype in ASCs, enhancing their ability to interact with and modulate M1-like macrophages. This shift supports the attenuation of pro-inflammatory responses, providing a strong rationale for the use of IFNγ-primed ASCs in the treatment of chronic inflammatory conditions [22,23,24,25,26,27].

Differently from inflammatory cytokines, hypoxic treatment of MSCs seems to stimulate primarily the secretion of functional factors involved in angiogenesis and tissue proliferation/regeneration [15,16,23]. In physiological cellular niches, oxygen tension is typically low, ranging between 2% and 8%. Replicating these hypoxic conditions *in vitro* has been shown to enhance stem cell survival, proliferation, and angiogenic capacity [28,29].

Despite preliminary evidence suggesting that MSCs, particularly when stimulated by inflammatory priming, which mimics *in vitro* the osteoarthritic microenvironment, can modulate signaling pathways related to immunomodulation, a clear identification of the therapeutic targets that can be regulated by these cells in OA is still missing. Here, we tried to address this question by integrating genomic, proteomic, and miRNome analyses to elucidate the potential mechanism of action of ASC-based treatments in OA.

## 2. Materials and Methods

### 2.1. Isolation and Expansion of Adipose-Derived Mesenchymal Stem Cells (ASCs)

Fresh human subcutaneous abdominal adipose tissue (Biopredic International, Saint-Grégoire, France) obtained from three donors was rinsed with phosphate-buffered saline containing antibiotics and digested with 0.075% type I collagenase for 40–90 min at 37 °C under gentle agitation. Following enzymatic digestion, the cell suspension was filtered through a 100 µm mesh and centrifuged to collect the stromal vascular fraction. The resulting pellet was resuspended in complete ASC growth medium (DMEM high glucose supplemented with 10% FBS, 1% HEPES, 1% sodium pyruvate, 1% penicillin-streptomycin, and L-glutamine). Cells were seeded at a density of 50,000 cells/cm^2^ and cultured at 37 °C in a humidified atmosphere with 5% CO_2_. At passage 1, ASCs were cryopreserved in 90% FBS containing 10% DMSO.

### 2.2. Priming of ASCs

For secretome collection, ASCs at 80–90% confluence were cultured as monolayers in serum-free DMEM-HG under various experimental conditions. For cytokine-primed conditions, the medium was supplemented with either 10 ng/mL IL1β (Miltenyi Biotec, Bergisch Gladbach, Germany) or 200 IU/mL IFNγ (Miltenyi Biotec, Bergisch Gladbach, Germany) and cells were incubated at 37 °C with 20% O_2_ and 5% CO_2_. For hypoxic conditioning (HYP), cells were cultured in serum-free DMEM-HG at 37 °C under 1% O_2_ and 5% CO_2_. These specific priming conditions were selected based on previous studies demonstrating their ability to induce enhanced immunomodulatory and regenerative MSC phenotypes. In particular, 10 ng/mL IL1β has been previously shown to activate reparative and immunomodulatory pathways in ASCs [19,20,21]. Likewise, 200 IU/mL IFNγ has been demonstrated to induce enhanced immunomodulatory activity when applied for MSC priming [23,24,25]. Regarding hypoxic priming, 1% O_2_, despite being lower than the oxygen levels typically reported within the knee joint (2–5% O_2_), was selected as an established condition frequently used in MSC studies to induce a strong hypoxic response and maximize the activation of HIF-dependent pathways [23,30,31].

Cells cultured without any inflammatory stimulus in normoxia were used as the control condition (Ctr). After 48 h, the supernatants were collected, centrifuged to remove cell debris, and stored at −80 °C until use.

### 2.3. RNA Extraction and Next-Generation Sequencing of ASCs

Total RNA was isolated using the RNeasy Mini kit (QIAGEN, Hilden, Germany). RNA quality and integrity were assessed with the Agilent 4200 TapeStation System (Agilent Technologies Ltd., Santa Clara, CA, USA). For RNA sequencing, libraries were generated starting from 1 µg of total RNA using the Illumina TruSeq Stranded Total RNA kit together with TruSeq RNA UD Indexes (Illumina, San Diego, CA, USA). In brief, ribosomal RNA was removed from the samples, and the remaining RNA was fragmented prior to first- and second-strand cDNA synthesis. Subsequently, adenylation of the 3′ ends of the double-stranded cDNA was performed, followed by ligation of Illumina indexed adapters. The resulting libraries were analyzed for size distribution and quality using the Agilent 4200 TapeStation System (Agilent Technologies Ltd., Santa Clara, CA, USA). Libraries were then quantified with the Qubit fluorometric assay (Invitrogen, Waltham, MA, USA), pooled at equimolar concentrations, and sequenced on an Illumina NextSeq™ 550 platform (Illumina, San Diego, CA, USA) using paired-end 2 × 76 bp reads according to the manufacturer’s protocol.

Raw sequencing data were processed in R (version 4.5.3), converting BCL files to FASTQ, trimming adapters, and aligning reads to the reference genome with STAR. Transcript abundances were quantified using RSEM, and lowly expressed genes were filtered. Normalization and dispersion estimation were performed with DESeq2, followed by differential expression analysis with Benjamini–Hochberg correction. Gene expression patterns were visualized with heatmaps and PCA and analyzed by volcano plots (*p* < 0.05; fold change > 1.5). Significantly deregulated genes for each priming condition were subjected to functional characterization with gene ontology enrichment analysis and pathway mapping, including protein–protein interaction network analysis, using the STRING database [32].

### 2.4. Exosome Isolation and Characterization

Secretome from both primed and unprimed cultures was processed for exosome (EXO) isolation by sequential centrifugation. The secretome was first spun at 300× *g* for 10 min to clear cellular debris, followed by centrifugation at 17,000× *g* for 25 min at 4 °C to remove remaining cells and large vesicular fragments. The supernatant was subsequently ultracentrifuged at 120,000× *g* for 90 min at 4 °C to collect the EXO fraction.

Exosome size distribution and concentration were determined using nanoparticle tracking analysis (NTA) on a NanoSight NS3000 system (Malvern Instruments Ltd., Malvern, UK). For each sample, three 60 s videos were acquired at a camera level of 16 under manually monitored temperature conditions. Particle data were then analyzed using the NTA software, version 3.3 (Build 3.3.104, Malvern Panalytical Ltd., Malvern, UK).

### 2.5. Mass Spectrometry Analysis

Protein digestion was performed using the Filter-Aided Sample Preparation (FASP) workflow with Vivacon centrifugal filters (10 kDa MWCO; Sartorius, Göttingen, Germany) as previously described [33]. Briefly, 2 mL of secretome was mixed with 100 mM Tris/HCl containing 8 M urea (pH 8.5) and incubated with 20 mM dithiothreitol (DTT; Thermo Fisher Scientific, Waltham, MA, USA) at 37 °C for 30 min. Proteins were then alkylated by adding 50 mM iodoacetamide (Thermo Fisher Scientific, USA) and incubating in the dark at room temperature for 5 min. The samples were washed twice with 100 mM Tris/HCl in 8 M urea (pH 8.0), after which protein digestion was initiated by adding LysC (0.2 μg; Promega, Madison, WI, USA) in 25 mM Tris/HCl, 2 M urea (pH 8.0) and incubating overnight. A subsequent digestion step was carried out for 4 h using trypsin (0.1 μg; Promega, USA) in 50 mM ammonium bicarbonate. The resulting peptides were purified using StageTip desalting (C18 resin, Sigma-Aldrich, St. Louis, MO, USA) and eluted in 60% acetonitrile with 0.1% formic acid [34]. Eluates were dried under vacuum (SpeedVac, Thermo Fisher Scientific, USA) and resuspended in 20 μL of 0.1% formic acid prior to LC-MS/MS acquisition.

Peptides (350 ng per injection) were analyzed using a Vanquish Neo UHPLC nanoLC system coupled to an Orbitrap Astral mass spectrometer (Thermo Fisher Scientific, USA). Samples were separated on a 75 µm × 150 mm DNV PEPMap Neo analytical column (Thermo Fisher Scientific, USA). Data-independent acquisition (DIA) was performed, beginning with a full MS1 scan from 380–980 *m*/*z*, followed by 299 consecutive DIA windows of 2 *m*/*z* width. MS1 spectra were acquired at a resolution of 240,000 with an AGC target of 5 × 10^6^ and a 5 ms maximum injection time. DIA scans were recorded at a fixed Astral resolution of 80,000, AGC target of 5 × 10^4^, and 3 ms maximum injection time, using HCD with a normalized collision energy of 25% and a scan range of 100–1000 *m*/*z*. DIA data were processed using DIA-NN (version 2.0.2) [35], employing a predicted spectral library generated from the Homo sapiens UniProt reference proteome (UP000005640_9606). Trypsin/LysC cleavage rules were applied, allowing up to two missed cleavages and requiring a minimum peptide length of six amino acids. Carbamidomethylation of cysteine and removal of the N-terminal methionine were set as fixed modifications, whereas methionine oxidation and N-terminal acetylation were considered variable. The peptide and protein false discovery rates (FDRs) were controlled at 0.01%. Protein quantification was carried out using label-free quantification (LFQ). Data processing was performed in Perseus (version 1.6.15.0) [36]. LFQ values were log_2_-transformed, and only protein groups quantified in at least 70% of samples within one experimental condition were retained for downstream analyses.

### 2.6. Cluster, Principal Component and Gene Ontology (GO) Analysis of Proteins

Protein abundance values were transformed into z-scores and subjected to hierarchical clustering to evaluate similarity among biological and technical replicates using Euclidean distance metrics and average link. Principal component analysis (PCA) was then applied to the log_2_-transformed protein expression matrix to assess variance within the dataset. Clustering and PCA procedures were carried out using Perseus software (version 2.0.11) [36]. Following volcano plot analysis (*p* < 0.05 and fold change > 1.5), significantly deregulated proteins identified for each priming condition were subjected to functional characterization, including protein–protein interaction network analysis, gene ontology enrichment, and pathway mapping using the STRING database [32].

### 2.7. RNA Extraction and Real-Time PCR Analysis of miRNAs

The expression profile of exosome-derived miRNAs was evaluated using the TaqMan Array Human MicroRNA Panels A and B v3.0 according to the manufacturer’s instructions (Thermo Fisher Scientific, USA). Total RNA was first extracted with the miRNeasy Mini Kit (Qiagen, Hilden, Germany). Subsequently, 300 ng of RNA was reverse-transcribed to generate single-stranded cDNA using the High Capacity RNA-to-cDNA Kit (Thermo Fisher Scientific, USA). Quantitative real-time PCR analysis of 754 human miRNAs was carried out using the QuantStudio 7 Pro Real-Time PCR System (Thermo Fisher Scientific, USA). Relative miRNA expression was determined using the 2^−ΔΔCT^ method, employing U6 small nuclear RNA as the internal reference control. In addition, principal component analysis (PCA) and hierarchical clustering were performed to identify sample groups sharing similar miRNA expression patterns.

### 2.8. miRNA Target Gene Prediction

Following volcano plot analysis (*p* < 0.05; fold change > 1.5), we performed functional enrichment analysis of the significantly deregulated miRNAs identified under each priming condition. Predicted target genes of these miRNAs were identified using the miRNet platform (https://www.mirnet.ca/faces/home.xhtml, accessed on 29 April 2026) [37] to construct protein–protein interaction (PPI) networks, allowing the elucidation of potential biological processes associated with the identified miRNAs.

### 2.9. Statistical Analysis

Statistical analyses were performed to identify significantly deregulated genes, proteins, and miRNAs among experimental conditions. Student’s *t*-test was used for normally distributed data, whereas the Mann–Whitney U test was applied for non-parametric data. For transcriptomic, proteomic, and miRNA analyses, statistical significance was combined with fold-change criteria to identify significantly deregulated features, which were subsequently used for volcano plot visualization and downstream enrichment analyses. Statistical significance was set at *p* < 0.05.

## 3. Results

### 3.1. Transcriptional Analysis of Primed ASCs

**Figure 1:**
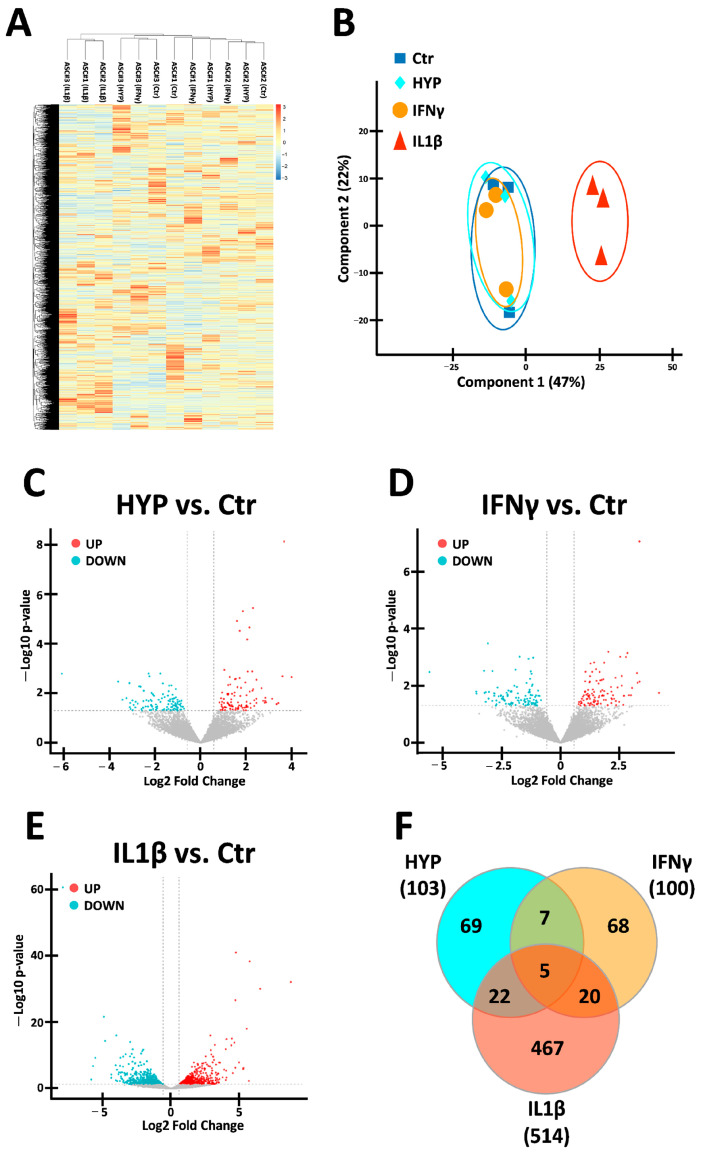
Transcriptomic profiling of primed ASCs. (**A**) Hierarchical clustering heatmap of differentially expressed genes across experimental conditions, showing distinct clustering of IL1β-primed ASCs compared to control (Ctr), hypoxia (HYP), and IFNγ-treated cells. (**B**) Principal component analysis (PCA) of global gene expression profiles, illustrating clear separation of IL1β-primed ASCs from all other groups, while HYP and IFNγ samples partially overlap with controls. (**C**–**E**) Volcano plots depicting differentially expressed genes in HYP vs. Ctr (**C**), IFNγ vs. Ctr (**D**), and IL1β vs. Ctr (**E**). Red and blue points indicate significantly upregulated and downregulated genes, respectively, while grey points represent non-significant changes (thresholds as defined in Methods). (**F**) Venn diagram showing the overlap of significantly upregulated genes among HYP, IFNγ, and IL1β conditions. IL1β priming induces the largest number of uniquely upregulated genes, with limited overlap between conditions, indicating a distinct transcriptional response compared to HYP and IFNγ treatments.

Transcriptomic profiling performed on three ASC donors identified a total of 12,442 expressed genes across all experimental conditions. Unsupervised hierarchical clustering and PCA revealed a clear separation of IL1β-primed ASCs from control (Ctr), hypoxia (HYP), and IFNγ-treated samples, which instead clustered closely together, indicating a limited transcriptional impact of HYP and IFNγ priming (Figure 1A,B).

Differential expression analysis confirmed these observations. IL1β priming induced a markedly broader transcriptional response, with 514 genes significantly upregulated compared to controls, whereas HYP and IFNγ treatments resulted in more limited gene expression changes relative to controls, with 103 and 100 significantly upregulated genes, respectively (Figure 1C–F). Moreover, Venn diagram analysis showed that the majority of upregulated genes were uniquely associated with IL1β stimulation (467 genes), with minimal overlap across conditions (Figure 1F), highlighting the dominant effect of IL1β in driving ASC transcriptional reprogramming.

**Figure 2:**
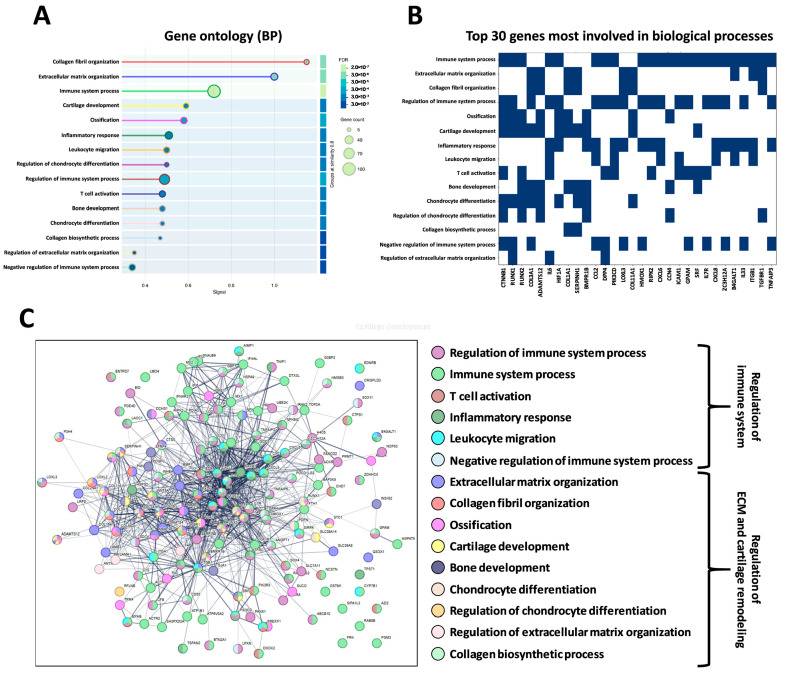
Functional characterization of IL1β-induced upregulated genes in ASCs. (**A**) GO enrichment analysis showing significant over-representation of immune regulation and cartilage/ECM remodeling processes. Biological Process (BP). (**B**) Top 30 contributing genes highlighting pleiotropic roles linking immune and extracellular matrix functions. (**C**) Protein–protein interaction network revealing a highly interconnected system with two main clusters: immune regulation and ECM/cartilage organization.

Through GO enrichment analysis of 514 upregulated genes, performed to investigate the biological relevance of IL1β-induced transcriptional changes, it was observed that enriched biological processes were mainly associated with immune regulation and cartilage remodeling (Figure 2A).

From these enriched categories, a subset of 164 genes associated with the selected pathways was extracted for further analysis: immune-related pathways included immune system process (n = 101 genes), regulation of immune system process (n = 65), inflammatory response (n = 32), negative regulation of immune system process (n = 24), T-cell activation (n = 21), and leukocyte migration (n = 19); whereas cartilage/ECM-related pathways included ossification and bone development (n = 39), extracellular matrix organization (n = 28), cartilage development (n = 16), collagen fibril organization (n = 13), chondrocyte differentiation (n = 18), and collagen biosynthetic process (n = 4). The analysis of the top 30 contributing genes, according to their involvement across enriched biological processes, demonstrated that several transcripts were involved in multiple pathways, suggesting pleiotropic functions linking immune modulation and tissue remodeling (Figure 2B). Protein–protein interaction network analysis of IL1β-induced upregulated genes revealed a highly interconnected gene network, with immune-related and extracellular matrix (ECM)/cartilage-associated pathways tightly integrated rather than acting as independent modules (Figure 2C).

Two major functional clusters can be observed. The first cluster was associated with immune system regulation, including inflammatory response, leukocyte migration, T cell activation, and regulation of immune signaling pathways. The second one encompassed ECM organization and cartilage-related processes, such as collagen fibril organization, collagen biosynthetic process, chondrocyte differentiation, and cartilage development. Overall, these findings indicate that IL1β priming induces a coordinated transcriptional program in ASCs that simultaneously engages immunoregulatory and cartilage regenerative pathways, supporting a dual functional phenotype relevant to osteoarthritis.

### 3.2. Proteomic Analysis of Primed ASCs

**Figure 3:**
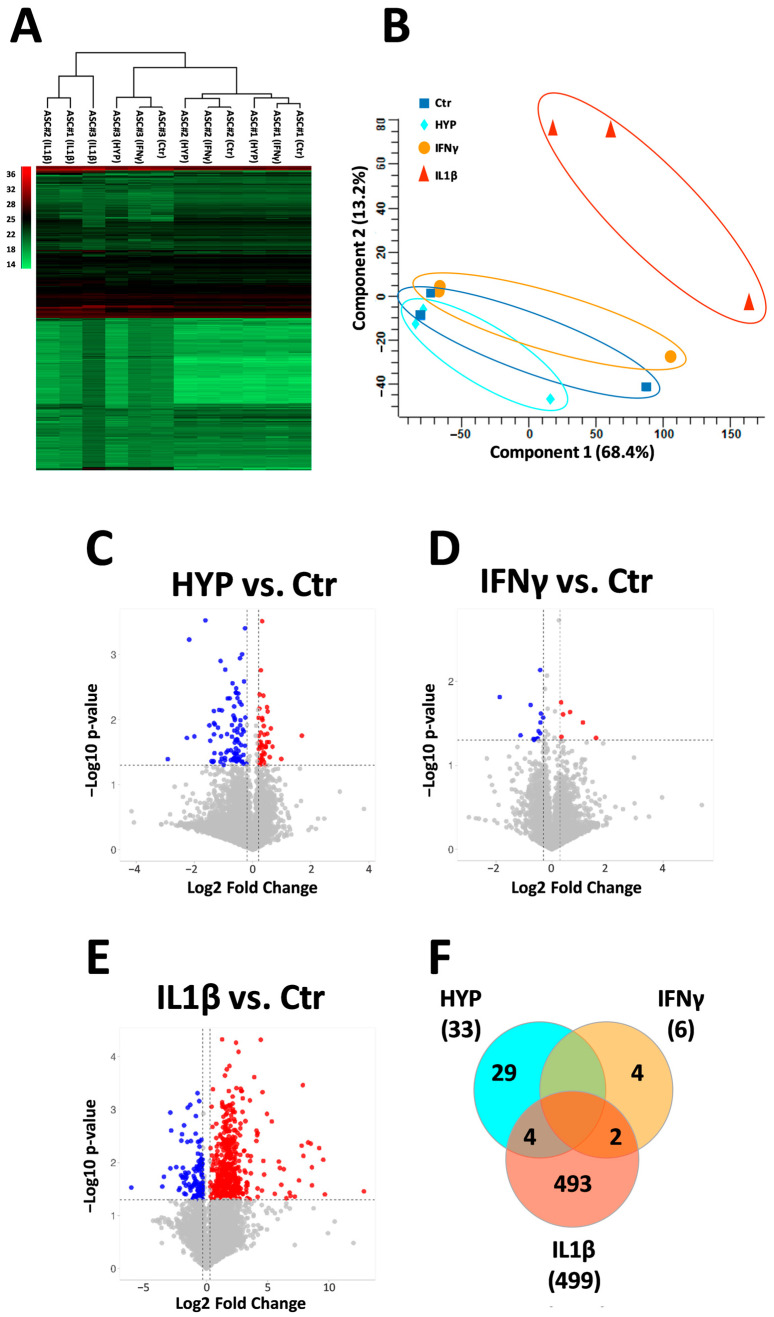
Proteomic analysis of ASC secretome under different priming conditions. (**A**) Hierarchical clustering of proteins showing limited changes under HYP and IFNγ, and a distinct IL1β-induced profile across donors. (**B**) PCA showing clear separation of IL1β-primed ASCs from all other conditions. (**C**–**E**) Volcano plots indicating minimal proteomic changes under HYP and IFNγ, and strong differential expression after IL1β priming. Red and blue points indicate significantly upregulated and downregulated genes, respectively, while grey points represent non-significant changes (thresholds as defined in Methods). (**F**) Venn diagram showing IL1β-specific upregulation of 493 proteins with few shared changes across conditions.

To investigate how different priming strategies modulate the paracrine therapeutic properties of ASCs, a proteomic analysis of secretome collected from three ASC donors cultured under control condition (Ctr) or subjected to hypoxia (HYP), IFNγ priming, or IL1β priming was conducted. A total of 7054 proteins were identified, where hierarchical clustering revealed that HYP- and IFNγ-primed ASCs displayed protein expression patterns closely resembling those of control cells across all donors, forming clusters indicative of limited proteomic remodeling (Figure 3A).

In contrast, IL1β priming induced a strong and consistent shift in the secretome across all biological replicates, resulting in three clearly segregated donor-specific clusters, each distinctly separated from the other priming conditions (Figure 3A). These observations were further supported by PCA (Figure 3B), where IL1β-primed ASCs formed a distant cluster in the PCA plot, while control, HYP-, and IFNγ-primed ASCs occupied a partially overlapping space, indicating markedly greater divergence induced by IL1β treatment. Differential expression analysis confirmed these trends. Volcano plots showed that HYP and IFNγ priming resulted in only limited changes in the ASC secretome compared to controls, with relatively few significantly upregulated or downregulated proteins (Figure 3C–E). Conversely, IL1β priming led to widespread proteomic remodeling, with a large number of proteins significantly deregulated compared to controls. In particular, as shown in the Venn diagram (Figure 3F), IL1β priming uniquely upregulated the expression of 493 proteins, while only a small subset of proteins was commonly regulated between IL1β and the other priming conditions (4 shared with HYP and 2 shared with IFNγ). These data indicated that IL1β priming elicits a robust and distinct secretory program in ASCs, unlike hypoxia and IFNγ, which induce comparatively limited changes.

**Figure 4:**
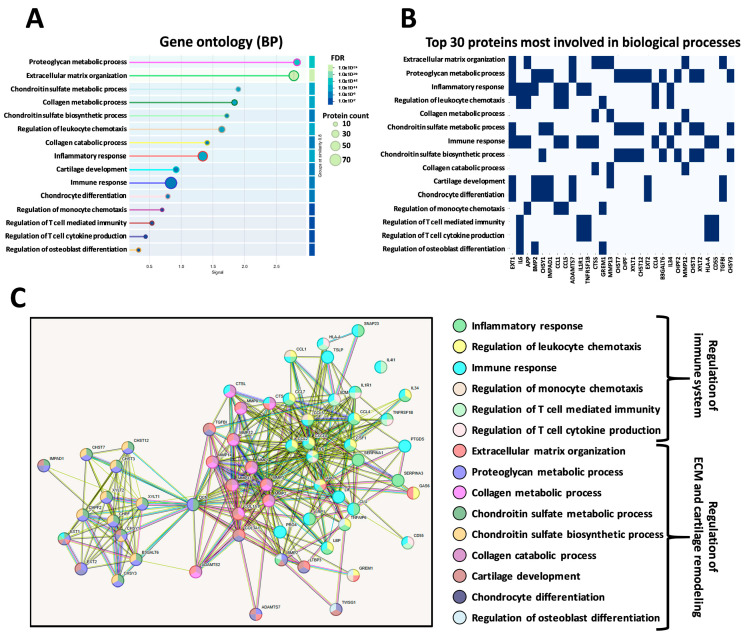
Functional analysis of IL1β-induced secreted proteins. (**A**) GO enrichment analysis of upregulated proteins showing two main clusters related to immune regulation and ECM/cartilage remodeling. Biological Process (BP). (**B**) Protein–process association matrix highlighting multifunctional proteins involved in both immunomodulatory and cartilage-related pathways. (**C**) Protein–protein interaction network revealing two densely interconnected functional modules (immune system and ECM/cartilage organization).

GO enrichment analysis on upregulated proteins was performed to further define the biological relevance of the protein network selectively induced by IL1β priming, revealing two major functional clusters, consistent with the RNA-Seq data and protein secretion data, primarily related to both immune system and cartilage remodeling (Figure 4A).

This dual functional signature was further supported by the protein–process association matrix, where several IL1β-induced proteins contributed simultaneously to both immunomodulatory, ECM-related and cartilage remodeling pathways (Figure 4B). The first cluster was associated with immune regulation, including inflammatory response, leukocyte and monocyte chemotaxis, and modulation of T cell-mediated immunity, indicating that IL1β priming shapes the immunoregulatory capacity of ASCs. The second cluster encompassed ECM organization and cartilage remodeling pathways, such as proteoglycan and chondroitin sulfate metabolic processes, collagen biosynthesis, chondrocyte differentiation, and cartilage development. STRING network analysis confirmed two main functional clusters with densely interconnected protein interactions, highlighting coordinated regulation rather than isolated protein changes (Figure 4C). These data are consistent with RNA-seq data, indicating that IL1β priming does not simply impact on the immunomodulatory properties of ASC secretome, but also promotes the secretion of factors involved in cartilage matrix turnover and regenerative remodeling, suggesting a mechanism by which IL1β-primed ASCs may simultaneously modulate inflammation and support joint tissue repair in OA.

The concordance between transcriptomic and proteomic data was supported by the identification of 77 genes consistently upregulated at both the mRNA and protein levels following IL1β priming (Supplementary Table S1), whose functional enrichment profiles closely reflected those obtained from each data set independently (Supplementary Figure S1).

### 3.3. miRNA Analysis of Primed ASCs

Whether the paracrine remodeling of ASCs induced by selected priming strategies also extended to the exosomal miRNome was then assessed through comparative profiling of 754 miRNAs in EXOs isolated from secretomes derived from three ASC donors.

**Figure 5:**
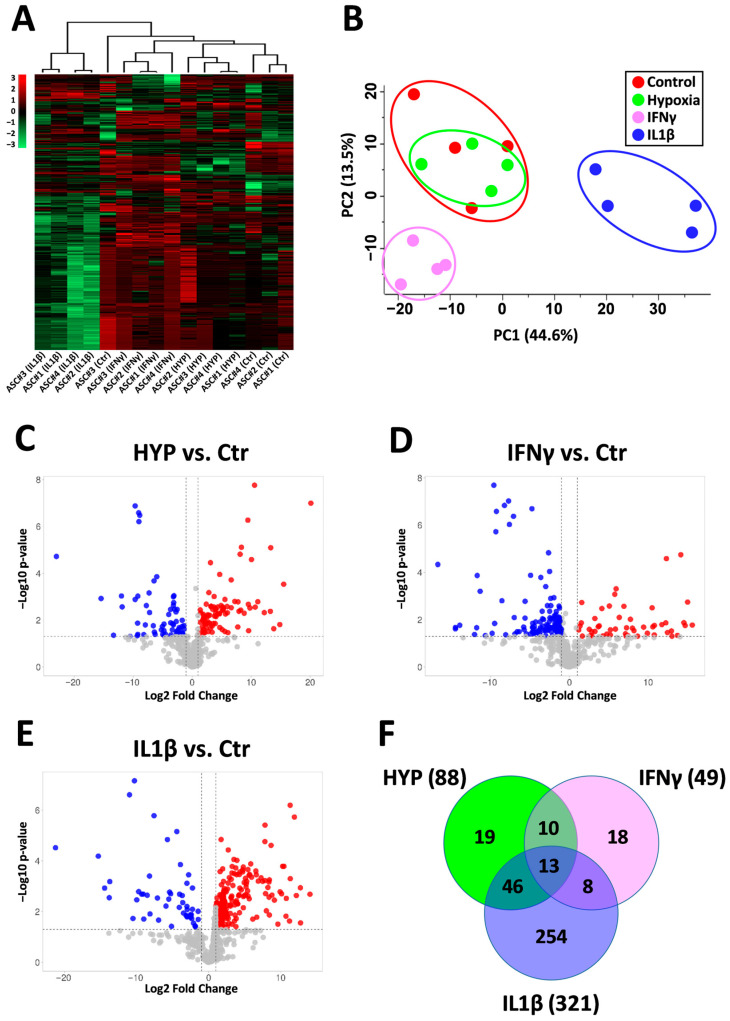
Exosomal miRNA profiling of ASCs under different priming conditions. (**A**) Hierarchical clustering of exosomal miRNAs showing close similarity between control, HYP-, and IFNγ-primed ASCs, and a distinct IL1β-specific profile. (**B**) PCA demonstrating clear separation of IL1β-primed samples from all other groups. (**C**–**E**) Volcano plots illustrating limited miRNA changes under HYP and IFNγ, and extensive differential expression after IL1β priming. Red and blue points indicate significantly upregulated and downregulated genes, respectively, while grey points represent non-significant changes (thresholds as defined in Methods). (**F**) Venn diagram showing minimal overlap among conditions, with IL1β inducing the largest set of uniquely upregulated miRNAs.

Hierarchical clustering of miRNA expression revealed that ASCs cultured under HYP or primed with IFNγ clustered closely with unstimulated controls, indicating minimal alterations in their exosomal miRNA signatures. In contrast, IL1β priming produced a distinct clustering pattern, with all IL1β-treated samples segregating clearly from the other groups (Figure 5A), suggesting that IL1β also drives a unique and consistent reprogramming of the ASC exosomal miRNA cargo across donors.

PCA confirmed this observation, with IL1β-primed ASCs occupying a clear separate and non-overlapping region of PCA space (Figure 5B). Volcano plot analysis identified distinct sets of significantly deregulated miRNAs in primed cells compared to controls (Figure 5C–E), and Venn diagram analysis revealed a limited overlap among priming conditions (Figure 5F), with only 13 miRNAs commonly upregulated across all treatments. The majority of upregulated miRNAs were uniquely associated with IL1β priming (254 miRNAs), while smaller condition-specific subsets were observed for hypoxia (19 miRNAs) and IFNγ (18 miRNAs), highlighting a strong priming-dependent miRNA signature.

**Figure 6:**
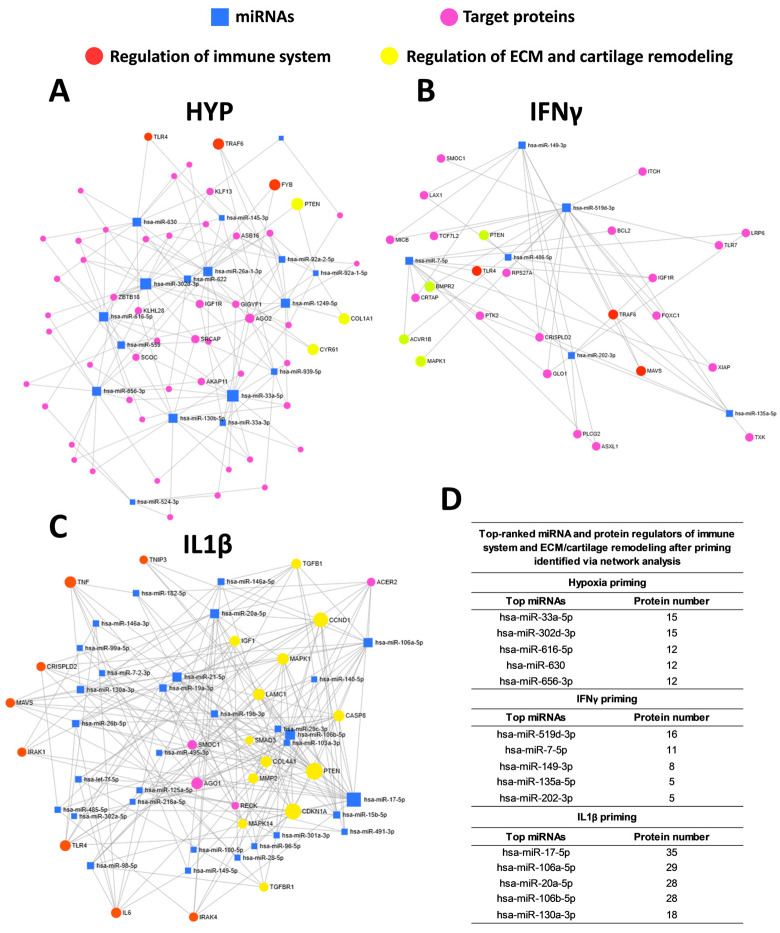
miRNA–target network analysis of ASC exosomal miRNAs under different priming conditions. (**A**,**B**) Hypoxia and IFNγ priming generate sparse miRNA–target networks with limited pathway convergence. (**C**) IL1β priming induces a dense and highly interconnected regulatory network linking miRNAs to genes involved in immune regulation and cartilage remodeling. (**D**) Top-ranked exosomal miRNAs and their corresponding number of target proteins involved in immune system regulation and ECM/cartilage remodeling, identified through network analysis under each priming condition (HYP, IFNγ, and IL1β).

To determine whether priming-induced miRNA-changes could mechanistically contribute to the dual immunomodulatory and regenerative responses, predicted miRNA–target interactions were analyzed using miRNet, revealing distinct interaction networks across priming conditions (Figure 6A–C).

Hypoxia and IFNγ priming generated relatively sparse interaction networks with limited convergence on the pathways of interest (Figure 6A,B). In contrast, IL1β priming produced a dense and integrated regulatory network linking multiple upregulated miRNAs which target proteins directly involved in both immune response modulation and cartilage remodeling (Figure 6C). Under hypoxic priming, the top-ranked miRNAs included hsa-miR-33a-5p and hsa-miR-302d-3p, each targeting up to 15 proteins, followed by hsa-miR-616-5p, hsa-miR-630 and hsa-miR-656-3p. IFNγ priming revealed a distinct profile, with hsa-miR-519d-3p showing the highest connectivity (16 target proteins), while hsa-miR-7-5p and hsa-miR-149-3p displayed moderate interaction networks. In contrast, IL1β priming resulted in a markedly higher regulatory complexity, with hsa-miR-17-5p targeting 35 proteins and several others (e.g., hsa-miR-106a-5p and hsa-miR-20a-5p) targeting more than 25 proteins.

**Figure 7:**
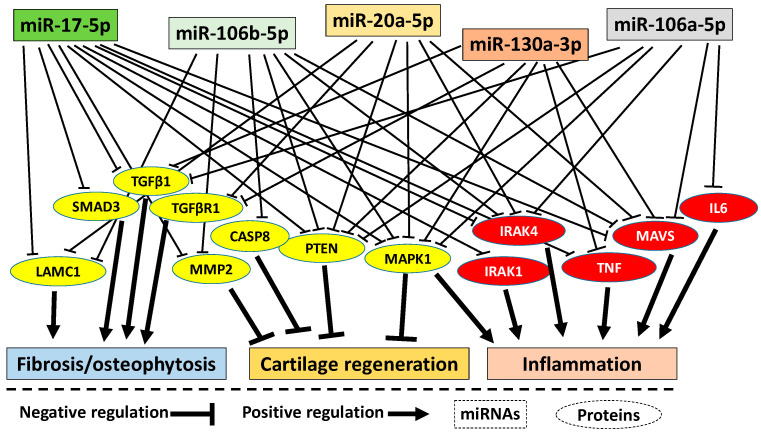
Functional targets of IL1β-associated exosomal miRNAs. IL1β-primed ASCs release miRNAs predicted to suppress key mediators of inflammatory signaling and fibrosis/osteophytosis pathways. In parallel, these miRNAs target regulators of cartilage regeneration and matrix turnover. Red indicates proteins involved in immune system regulation, whereas yellow indicates proteins involved in ECM/cartilage remodeling.

Specifically, IL1β-associated miRNAs were predicted to suppress key mediators of both inflammatory signaling and the induction of fibrosis and osteophytosis, including IL6, MAVS, IRAK1, IRAK4, TNF, TGFβ1, TGFβR1, SMAD3, and LAMC1 (Figure 7).

This regulatory profile is consistent with an enhanced immunosuppressive capacity and inhibition of fibrotic responses and pathological structural remodeling associated with osteophyte formation. Concurrently, these miRNAs were predicted to target proteins involved in the inhibition of cartilage regeneration, such as MAPK1, PTEN, CASP8, and MMP2, which play critical roles in cartilage matrix turnover (Figure 7). These findings indicate that IL1β priming uniquely reprograms ASC-derived exosomal miRNAs toward a coordinated regulation of inflammation resolution and cartilage repair.

## 4. Discussion

This study provides an integrated multi-omics analysis of human ASCs, showing that IL1β priming, in contrast to hypoxia and IFNγ, induces a profound reprogramming of their transcriptional, secretory, and regulatory profiles. This shift translates into an enhanced potential to modulate immune responses while supporting cartilage regeneration, two key processes in OA pathophysiology [38,39,40].

At the transcriptional level, IL1β priming emerged as the dominant driver of ASC reprogramming, inducing a substantially broader and largely distinct gene expression signature compared to other priming conditions. Functional enrichment and network analyses indicated that these changes are not restricted to isolated pathways, instead reflecting tightly interconnected programs linking immune regulation with ECM organization and cartilage-related processes. This coordinated transcriptional landscape suggests that IL1β induces a dual functional phenotype in ASCs, simultaneously targeting inflammation and tissue remodeling.

This finding is particularly relevant in OA because inflammation and cartilage degeneration are tightly interconnected processes rather than independent pathological events [41]. Therefore, a therapeutic strategy simultaneously targeting both mechanisms may provide greater efficacy than approaches acting exclusively on inflammation or tissue regeneration.

Importantly, this transcriptional reprogramming was reflected at the proteomic levels. IL1β priming induced a marked remodeling of the ASC secretome, with numerous uniquely upregulated proteins enriched in pathways associated with immune regulation, immune cell chemotaxis, ECM organization, and cartilage development. Together, these findings indicate that IL1β-driven transcriptional programs are effectively translated into a functional secretory phenotype, strengthening the biological relevance of this priming strategy and its potential to enhance the therapeutic efficacy of ASCs.

In parallel, exosomal miRNA profiling revealed that IL1β priming generates a distinct and extensive miRNA signature, characterized by a high number of uniquely upregulated miRNAs and a regulatory network targeting key mediators of inflammation and cartilage remodeling.

The integration of transcriptomic, proteomic, and miRNA data supports a multi-layered regulatory framework in which IL1β priming enhances ASC functionality. The use of a multi-omics strategy represents a major strength of the present study because it enables the investigation of regulatory mechanisms across multiple biological layers. This integrated approach reduces the limitations associated with single-omics analyses and provides a more comprehensive understanding of ASC functional reprogramming.

Rather than acting through isolated molecular changes, IL1β appears to orchestrate coordinated regulatory networks that simultaneously dampen pro-inflammatory signaling, limit fibrotic and osteophytic processes, and promote cartilage regenerations. This systems-level reprogramming supports the hypothesis of a superior immunomodulatory and regenerative potential of IL1β-primed ASCs [19,21].

In contrast, hypoxia and IFNγ priming induced modest alterations across all omics layers, with limited impact on both secretome composition and miRNA-mediated regulatory networks. The relatively limited effects observed after IFNγ and hypoxic priming suggest that the biological impact of priming may be highly context-dependent and influenced by the specific therapeutic target [15]. While hypoxia may preferentially support angiogenic and proliferative programs [42,43,44] and IFNγ may predominantly affect selected immune pathways without significantly engaging regenerative processes [22,26,27], these stimuli did not induce the broad and coordinated molecular response observed with IL1β. These observations highlight the importance of priming context in determining ASC functional outcomes. However, one limitation of the present study is that priming conditions were evaluated only individually and without testing any combination. Although this approach enabled the identification of stimulus-specific molecular signatures, combinatorial strategies such as IL1β plus hypoxia or IFNγ may more closely reproduce the multifactorial OA microenvironment and could potentially generate synergistic therapeutic effects, thus representing an interesting opportunity for the further optimization of ASC-based therapeutic products.

From a translational perspective, this work provides mechanistic insight into how inflammatory priming can be leveraged to enhance ASC-based therapies for OA. Moreover, the identification of specific proteins and miRNAs involved in these processes may provide candidate biomarkers or therapeutic targets for optimizing cell-based interventions. The multi-omics evidence, consistent with previous literature [21,45] and supported by *in vitro* functional immunomodulatory data [19], suggests that IL1β-primed ASCs may represent a more effective strategy for restoring joint homeostasis by simultaneously targeting immune dysregulation and cartilage degeneration. Previous studies have shown that IL1β priming enhances MSC immunomodulatory properties and increases the secretion of factors involved in cartilage protection [21]. However, these studies mainly focused on individual molecular layers or specific functional assays. In contrast, our study integrates transcriptomic, proteomic, and exosomal miRNA analyses, providing a systems-level characterization of ASC responses and revealing coordinated regulatory networks linking immune regulation and tissue remodeling. In particular, we previously demonstrated that ASCs can modulate macrophage polarization and T cell survival, fostering an overall anti-inflammatory environment [20]. On the other hand, the long-term therapeutic efficacy and safety of IL1β-primed ASCs remain to be validated in in vivo OA models. This is essential to confirm that the functional effects associated with the molecular signatures identified through omics integration and predictive network analyses are indeed reflected in the therapeutic performance of this cell-based product. Nevertheless, the therapeutic efficacy of these approaches is also expected to be influenced by OA stage and patient-specific molecular features, considering the highly heterogeneous and multifactorial nature of the disease. The integration of advanced molecular profiling approaches, including proteomic and miRNomic characterization of patient-derived biological fluids, with bioinformatic analyses may support the identification of potential therapeutic matches between specific MSC-based products and distinct OA molecular phenotypes, paving the way toward more personalized regenerative medicine strategies. Moreover, future studies could explore whether the combination of tailored priming approaches with more site-specific MSC sources, such as infrapatellar fat pad-derived ASCs, may further enhance the tissue-targeted therapeutic potential of MSC-derived secretomes and extracellular vesicles.

Collectively, these findings support a model in which IL1β priming impacts ASC functionality through coordinated transcriptomic, proteomic, and miRNA-mediated reprogramming, thus coupling immune modulation with tissue remodeling pathways relevant to osteoarthritis.

## 5. Conclusions

This multi-omics study identifies IL1β priming as the most effective strategy, among those tested, to enhance the therapeutic profile of human ASCs, both in terms of immune regulation and cartilage remodeling. In contrast, hypoxia and IFNγ induced only limited and less coordinated molecular responses across omics layers. By integrating transcriptomic, proteomic and exosomal miRNA analyses of secretome, IL1β-primed ASCs display a distinct functional profile characterized by coordinated immunomodulatory and regenerative properties. Overall, these findings highlight the critical role of inflammatory priming in shaping ASC functionality and provide a molecular framework to optimize ASC-based therapies for osteoarthritis.

## Data Availability

Other original data presented in the study are openly available at the following link: https://osf.io/uzrqa/overview?view_only=305ff4984ee346f9860771530d0856ba (accessed on 16 April 2026).

## Supplementary Materials

The following supporting information can be downloaded at: https://www.mdpi.com/article/10.3390/cells15121056/s1, Table S1: Genes consistently upregulated at both the mRNA and protein levels following IL1β priming; Figure S1: Functional convergence of transcriptomic and proteomic profiles following IL1β priming. (A) GO enrichment analysis. (B) Protein–protein interaction network.

## Abbreviations

The following abbreviations are used in this manuscript:

ASC	Adipose-derived mesenchymal Stem Cell
Ctr	Control
DIA	Data Independent Acquisition
DMSO	Dimethyl sulfoxide
ECM	Extracellular Matrix
EXO	Exosome
FBS	Fetal Bovine Serum
FASP	Filter-Aided Sample Preparation
GO	Gene Ontology
HYP	Hypoxia
IFNγ	Interferon gamma
IL1β	Interleukin 1 beta
IRAK	Interleukin-1 Receptor-Associated Kinase
LAMC1	Laminin subunit gamma 1
LC-MS/MS	Liquid Chromatography tandem mass spectrometry
LFQ	Label-Free Quantification
MAVS	Mitochondrial Antiviral signaling protein
MMP2	Matrix Metalloproteinase 2
MSCs	Mesenchymal Stem Cells
NTA	Nanoparticle Tracking Analysis
OA	Osteoarthritis
PCA	Principal Component Analysis
PTEN	Phosphatase and Tensin homolog
RNA-Seq	RNA Sequencing
SMAD3	SMAD family member 3
STRING	Search Tool for the Retrieval of Interacting Genes/Proteins
TNF	Tumor Necrosis Factor
TGFβ	Transforming Growth Factor beta
TGFβR1	Transforming Growth Factor beta Receptor 1
U6	Small nuclear RNA U6
