# Supplementary material for: A Multi-Omics Approach Reveals Interleukin 1 Beta Priming as a Key Driver of Immunomodulatory and Regenerative Programs in Adipose-Derived Stem Cells for Osteoarthritis Therapy
Source: Cells. 2026 Jun 9;15(12):1056. doi: 10.3390/cells15121056 (PMC13297184; doi:10.3390/cells15121056)
Supplement: Supplementary file 1 [file cells-15-01056-s001.zip › cells-4321652-supplementary.pdf]

Table S1. genes consistently upregulated at both the mRNA and protein levels following IL1β priming.

Protein	Gene
ACO1	ACO1
ADAM19	ADAM19
ADAMTS4	ADAMTS4
ANGPT1	ANGPT1
ANTXR1	ANTXR1
B2M	B2M
BID	BID
C1S	C1S
C3	C3
CCL2	CCL2
CD55	CD55
COL16A1	COL16A1
COL1A1	COL1A1
COL3A1	COL3A1
COL5A1	COL5A1
COL7A1	COL7A1
CPD	CPD
CREG1	CREG1
CTSS	CTSS
CXCL1	CXCL1
CXCL2	CXCL2
CXCL3	CXCL3
CXCL5	CXCL5
DCBLD1	DCBLD1
DCHS1	DCHS1
DNAJB9	DNAJB9
DPP4	DPP4
FAP	FAP
FGF2	FGF2
FNDC1	FNDC1
GALNT2	GALNT2
GPC6	GPC6
HSPA13	HSPA13
HSPG2	HSPG2
HYOU1	HYOU1
ICAM1	ICAM1
IL11	IL11
IL6	IL6
IL7R	IL7R
ITGA1	ITGA1
ITGAV	ITGAV
ITGB1	ITGB1
LACC1	LACC1

LAMA1	LAMA1
LAMB3	LAMB3
LGI2	LGI2
LIF	LIF
LOXL3	LOXL3
LRP8	LRP8
LRRC15	LRRC15
LSAMP	LSAMP
MMP1	MMP1
MMP14	MMP14
MMP2	MMP2
MMP3	MMP3
MPZL1	MPZL1
MX2	MX2
NCSTN	NCSTN
OSMR	OSMR
PAPPA	PAPPA
PDGFC	PDGFC
PHLDA1	PHLDA1
PTK7	PTK7
PTPRN	PTPRN
PXDN	PXDN
QSOX1	QSOX1
ROBO1	ROBO1
SDC4	SDC4
SIRPA	SIRPA
SLC39A14	SLC39A14
TFRC	TFRC
TMEM132A	TMEM132A
TNFAIP3	TNFAIP3
TNFAIP6	TNFAIP6
TNIP1	TNIP1
TPST1	TPST1
UXS1	UXS1

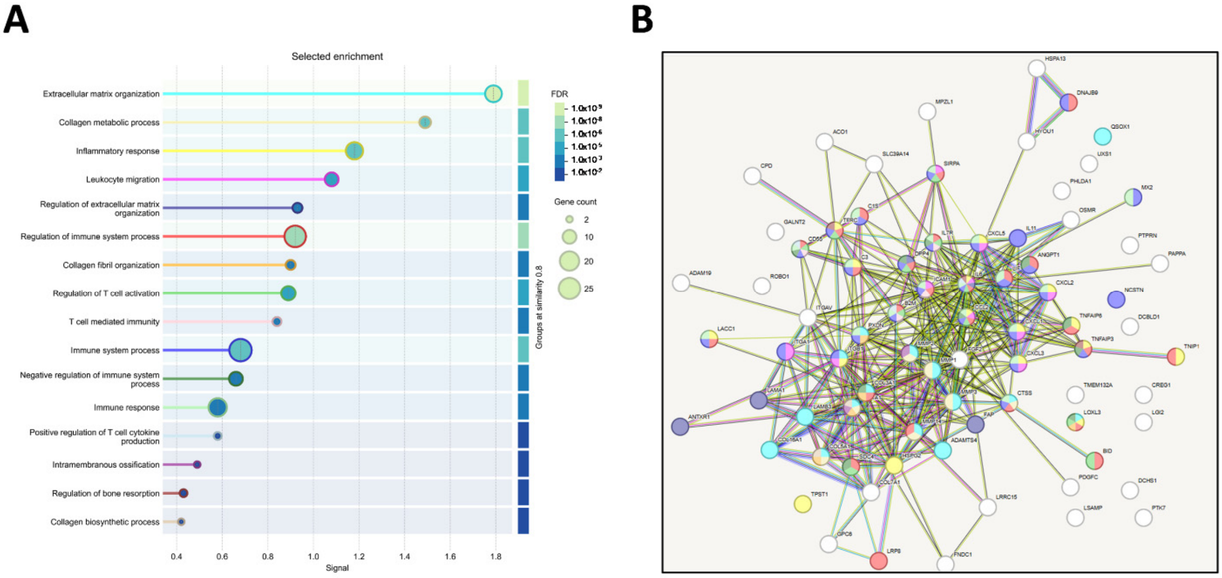

Figure S1. Functional convergence of transcriptomic and proteomic profiles following IL1β priming. (A) GO enrichment analysis. (B) Protein–protein interaction network.
